# Assessment of the state of pharmacovigilance in the South-South zone of Nigeria using WHO pharmacovigilance indicators

**DOI:** 10.1186/s40360-018-0217-2

**Published:** 2018-05-31

**Authors:** Abimbola O. Opadeyi, Annie Fourrier-Réglat, Ambrose O. Isah

**Affiliations:** 1 Department of Clinical Pharmacology and Therapeutics, University of Benin, Benin-City, Edo State Nigeria; 2 Department of Medicine, University of Benin Teaching Hospital, Benin-City, Nigeria; 3 Inserm, Bordeaux Population Health Research Center, team, Pharmacoepidemiology, University Bordeaux, UMR 1219, F-33000 Bordeaux, France; 4 Bordeaux PharmacoEpi, INSERM CIC1401, F-33000 Bordeaux, France; 5 CHU de Bordeaux, Pôle de santé publique, Service de Pharmacologie médicale, F-33000 Bordeaux, France; 6 National Drug Safety Advisory Committee, National Agency for Food and Drug Administration and Control, Federal Ministry of Health, Abuja, Nigeria

**Keywords:** Pharmacovigilance, Adverse drug reaction reporting, Nigeria, Tertiary hospitals

## Abstract

**Background:**

WHO pharmacovigilance indicators have been recommended as a useful tool towards improving pharmacovigilance activities. Nigeria with a myriad of medicines related issues is encouraging the growth of pharmacovigilance at peripheral centres. This study evaluated the status of pharmacovigilance in tertiary hospitals in the South-South zone of Nigeria with a view towards improving the pharmacovigilance system in the zone.

**Methods:**

A cross-sectional descriptive survey was conducted in six randomly selected tertiary hospitals in the South-South zone of the country. The data was collected using the WHO core pharmacovigilance indicators. The language of assessment was phrased and adapted in this study for use in a tertiary hospital setting. Data is presented quantitatively and qualitatively.

**Results:**

A total of six hospitals were visited and all institutions had a pharmacovigilance centre, only three could however be described as functional or partially functional. Only one centre had a financial provision for pharmacovigilance activities. Of note was the absence of the national adverse drug reaction reporting form in one of the hospitals. The number of adverse drug reaction reports found in the databases of the centres ranged from none to 26 for the previous year and only one centre had fully committed their reports to the National Pharmacovigilance Centre. There were few documented medicines related admissions ranging from 0.0985/1000 to 1.67/1000 and poor documentation of pharmacovigilance activities characterised all centres.

**Conclusion:**

This study has shown an urgent need to strengthen the pharmacovigilance systems in the South-South zone of Nigeria. Improvement in medical record documentation as well as increased institutionalization of pharmacovigilance may be the first steps to improve pharmacovigilance activities in the tertiary hospitals.

**Electronic supplementary material:**

The online version of this article (10.1186/s40360-018-0217-2) contains supplementary material, which is available to authorized users.

## Background

Pharmacovigilance in Nigeria commenced in the late 80s and early 90s initially in a tertiary hospital with some preparatory activities at the national level prior to its admission into the WHO program for international drug monitoring (PIDM) in 2004 [1, 2]. It has sustained its activities through active training of healthcare workers, sensitisation campaigns using print and electronic media about medicine safety issues to health care workers and the public [3]. It has also carried out active surveillance through the cohort event monitoring on adverse reactions to antimalarials (artemisinin-based combination therapy) [4]. There has also been the introduction of electronic devices to reduce substandard and falsified medical products which is a major contributor to adverse drug reactions in our setting.

The growth of pharmacovigilance in Nigeria has been propelled by a number of factors including the establishment of the regulatory agency (National Agency for Food and Drug Administration and Control – NAFDAC) by Decree 15 of 1993 (as amended) now cited as Act Cap N1 laws of the Federal Republic of Nigeria 2004, the formulation of the Nigerian National Drug Policy in 2005 [5]. This was further clarified by the introduction of the Nigerian pharmacovigilance policy document in 2012 firmly positing drug safety in national discussion [6]. The actualization of some of these goals has recorded significant progress with the formation of the zonal centres to cover the six geo-political zones in the country in 2012 [7].

Pharmacovigilance has a wide scope with increasing product concerns. The main focus in the Nigeria context has been on adverse drug reactions, substandard and falsified medical products [3, 8–10]. Other areas yet to be fully addressed include medication errors [11], lack of effectiveness reports, acute and chronic poisoning [12, 13], assessing drug related mortality as well as abuse and misuse of medicines [3, 9]. The determination of the burden of these various problems has been poor as the major challenge to the growth of pharmacovigilance in Nigeria has been that of underreporting as seen worldwide [14–16].

Reporting of drug safety concerns by health-workers in Nigeria is voluntary and the reasons for under-reporting are partly due to fear of litigation, poor understanding of the subject matter, feeling that the “known” Adverse Drug Reactions (ADRs) need not be reported, time constraints and cumbersome reporting processes [17–21]. Lack of appropriate structures and deficient processes at the institutional level may also contribute to the poor reporting rate as found in some studies [17, 21–23].

WHO advocates regional centres as an effective way of enhancing pharmacovigilance activities [24] as observed in some areas of the world where this has been found to improve the number and quality of reports [25, 26].

The aims of the creation of the zonal centres was to decentralize the activities of the National Pharmacovigilance Centre (NPC), e.g. distribution of ADR forms and collection of the Individual Case Safety Reports (ICSRs) from reporters and perform preliminary evaluation with prompt reporting, also transmission of acknowledgements and feedback information to the reporters and dissemination of information from the national centre to the patients and health care workers. Furthermore, they were created to monitor the progress of pharmacovigilance activities at institutional levels as well as support the training and capacity building for pharmacovigilance in the areas of their jurisdiction [6]. These measures would further increase awareness about pharmacovigilance and instil a sense of ownership among the stakeholders regarding pharmacovigilance activities as well as bring closer to the reporters a centre close to their practice.

Currently, the assessment of pharmacovigilance had been largely done at the national level using various tools including evaluating the attainment of minimum requirements for a national centre with interviews of focal persons [27], and recently the use of the Indicator based Pharmacovigilance Assessment Tool (IPAT) indicators [28]. The more recent introduction of the WHO pharmacovigilance indicators provides an opportunity to assess pharmacovigilance activities at the national centres [29]. These indicators targeted at the national centres perform self-evaluation and also identify areas that require intervention. This approach may be applicable to zonal centres and its components which feed data to the national centres. It may also be most appropriate to identify problems at sub-national levels requiring attention [30].

The status of the pharmacovigilance system in the tertiary centres is presently unknown as the WHO indicators and related metrics for evaluating these centres have just been recently released [29] and there is little or no data on the effectiveness and functionality of these centres at this time. Furthermore, the involvement of these centres in this self-appraisal will further facilitate their participation in measures to remedy identified deficiencies with a view towards improving the quantity and quality of adverse drug reaction reports and other areas in pharmacovigilance. This study intends to assess the status of pharmacovigilance structure, processes, outcomes and impact in the South–South zone of Nigeria using the newly introduced WHO pharmacovigilance indicators.

## Methods

### Study setting and design

This study was carried out in the South-South Zone of Nigeria which is located in the coastal region of Nigeria. It comprises six states namely Akwa-Ibom, Bayelsa, Cross Rivers, Delta, Edo and Rivers with a population of 21,014, 655 million persons (Nigeria national census 2006). Health care professionals in all tiers of hospitals in this zone could send their reports either directly or through the zonal pharmacovigilance centre for onward transmission to the national centre. The South-South zonal pharmacovigilance centre is domiciled in the University of Benin Teaching Hospital, a tertiary hospital for research and learning.

In Nigeria, heath care is delivered at three levels: primary, secondary and tertiary. Tertiary care hospitals provide the highest level of care and serve as referral centres for the secondary and primary centres. Furthermore, there are three main types of tertiary centres. Firstly: the teaching hospitals, which provide teaching (to most cadres in the health professions at undergraduate and postgraduate levels for medical, nursing, pharmacy students etc.) as well as for research and health care services. Secondly: Federal Medical Centres which are mainly for health care services as well as providing residency training in some departments and lastly the specialty hospitals which focuses on particular disease entities of public health importance such as neuro-psychiatric hospitals, orthopaedic hospitals and ophthalmic hospitals among others.

This study was directed at the teaching hospitals because they provide the widest access to all patients with an inclusiveness of all cadres of health care workers. In the South-South zone there are eight teaching hospitals, seven are government owned, and one privately owned.

Eligibility criteria: teaching hospitals were used to ensure inclusiveness of all clinical disciplines and staff complement. All six states in the zone were represented by a teaching hospital. An institutional approval was required from the Chief Medical Director / Management prior to inclusion in the study. The study was subsequently carried out in 6 tertiary health institutions selected through simple random sampling in the various states namely:

- University of Benin Teaching Hospital Benin-City, Edo State, (UBTH).
- Delta State University Teaching Hospital Oghara, Delta State, (DELSUTH).
- Niger Delta University Teaching Hospital Okolobri, Bayelsa State, (NDUTH).
- University of Port Harcourt Teaching Hospital, Port Harcourt, Rivers State, (UPTH).
- University of Uyo Teaching Hospital, Uyo, Akwa- Ibom State, (UUTH).
- University of Calabar Teaching Hospital, Calabar Cross-River State, (UCTH).

Prior to visiting the study sites for data collection, ethical approval was obtained from the research and ethics committee of each of the selected tertiary hospitals. Furthermore, the heads of the institution were contacted for approval and access to the pertinent data. The focal persons in charge of pharmacovigilance in the institution provided answers for the indicator assessments.

### Data collection

The data were obtained using a modified WHO data collection form for pharmacovigilance indicators [29] by one of the researchers through interviews of the focal person for pharmacovigilance or the pharmacovigilance committee. The components of this form included *the background information*, *structural indicators, process indicators, outcome/impact indicators.* The phrasing of the assessment questions was adapted to address the tertiary hospital setting (Additional file 1).

The *background information* collected characteristics of the hospitals: teaching hospital staff strength, i.e. number of post registration health professionals in different categories: doctors, nurses, pharmacists, specialist disposition, average out-patient attendance over the last year, total number of beds in the hospital.

The *structural indicators* assessed the existence of key pharmacovigilance structures, systems and mechanisms in any of the settings studies. It details the basic infrastructure needed to enable good pharmacovigilance activities. It assesses the enabling environment needed for pharmacovigilance activities.

The *process indicators* assessed the degree of pharmacovigilance activities in the various centres. It focussed on the processes that describe the collection, collation, analysis and evaluation of ADR reports. The factors influencing these processes were also considered. These measures were assessed directly or indirectly.

The *outcome/impact indicators* measure the extent of realization of the pharmacovigilance objectives. The hospital records used in assessing the outcome/impact indicators include admission and discharge registers, death registers, International coding of disease registers where available. Other requested details were: the total number of outpatient visits in the previous year, the morbidity and mortality statistics of each institution for the previous year (to include the disease statistics of admitted and deceased persons). Furthermore, to compute the duration of hospital stay, the crude estimates of the duration of admission of patients with serious adverse reactions who were hospitalised was calculated from the adverse drug reaction reports obtained for the previous year.

### Data analysis

Analysis was both qualitative and quantitative. All hospitals participating in the study were described according to each indicator. The core Structural indicators are qualitative indicators with categorical data analysed descriptively. The presence or absence of the parameter measured was described for each institution.

Analysis of the core Process and Outcome Indicators are quantitative indicators reflecting rates of reports and actual numbers. They were calculated using frequencies and absolute numbers as dictated by the indicator. The data was analysed with descriptive statistics using Microsoft excel 2007.

## Results

All six institutions were visited and the focal Pharmacovigilance persons or committees interviewed following a meeting with the various heads of the institutions. The teaching hospitals in this study are all government owned and serve as referral centres to the primary and secondary tier hospitals. However, they are of varying sizes in terms of bed and staff complement. The demographic characteristics of the institutions at the commencement of the study late January to mid-March 2016 were as follows (Table 1).

**Table 1:** Characteristics of the tertiary teaching hospitals in the South-South Zone^a^

Characteristic	UCTH	UUTH	UPTH	NDUTH	DELSUTH	UBTH
Number of beds	610	499	782	148	250	701
Approximate number of health care workers (post registration)	1141	739	1028	253	532	1219
Consultant Clinicians	146	86	179	85	65	200
Doctors	359	124	210	53	150	335
Nurses	580	417	600	105	300	660
Pharmacists	56	19	39	10	17	24
Out-patient attendance in the previous year (2015)	81,624	114,523	114,277	32,906	22,540	179,255
Number of in-patient hospital admissions (2015)	7171	9679	10,145	2548	No data	11,324

^a^*UBTH* University of Benin Teaching Hospital Benin-City, Edo State, *UCTH* University of Calabar Teaching Hospital, Calabar Cross-River State. *UPTH* University of Port Harcourt Teaching Hospital, Port Harcourt, Rivers State, *UUTH* University of Uyo Teaching Hospital, Uyo, Akwa- Ibom State. *DELSUTH* Delta State University Teaching Hospital Oghara, Delta State, *NDUTH* Niger Delta University Teaching Hospital Okolobri, Bayelsa State

### Core structural indicators

Responses were obtained from the interviewed personnel for the assessment questions of the 10 structural indicators for all the institutions studied. Three of the 6 institutions had a standardised functional accommodation for pharmacovigilance activities while 1 had non functional rooms and 2 had none. Only one hospital had regular financial provisions for pharmacovigilance. The secretariat in 4 centres had a full time staff to carry out pharmacovigilance activity while 2 had part time staff. Of note was the availability of an institutionalized ADR reporting form in one of the six centres (DELSUTH) while a centre neither had copies of the national nor local forms available. There were no standard forms available which addressed the subset of assessment questions covering the scope of pharmacovigilance in all of the centers (Table 2).

**Table 2:** Analysis of WHO Core Pharmacovigilance Structural Indicators of the six tertiary hospitals in the South-South zone of Nigeria

Indicator Item	Assessment	UCTH	UUTH	UPTH	NDUTH	DELSUTH	UBTH	Hospitals with positive answers (n)
CST1	Presence of pharmacovigilance centre/department /unit with a standard accommodation.	Yes	No	No	No	Yes	Yes	3
CST2	Availability of a copy of the Nigerian pharmacovigilance policy	Yes	No	No	Yes	Yes	Yes	4
CST3	Presence of Institutional Drug Therapeutic Committee	Yes	No	No	Yes	Yes	Yes	4
CST4	Availability of regular financial provision for the pharmacovigilance Centre.	No	No	No	No	No	Yes	1
CST5	Availability of human resources to carry out functions of Pharmacovigilance Centre.	Yes	Yes	Yes	Yes	Yes	Yes	6
CST6	Availability of standard ADR reporting form in the institution.	Yes	Yes	No	Yes	Yes	Yes	5
	CP6a-e: Availability of relevant fields in standard ADR reporting form for a) medication error, b) counterfeit/substandard medicines, c) therapeutic ineffectiveness, d) suspected misuse, abuse, dependence on medicines, e) general public.^a^	No	No	No	No	No	No	0
CST7	A process is in place for collection, recording and analysis of ADR reports	Yes	No	No	No	Yes	Yes	3
CST8	Incorporation of pharmacovigilance into the orientation programme curriculum of newly employed health care professionals	No	No	No	No	No	Yes	1
	CST8a: for Medical doctors	No	No	No	No	No	Yes	1
	CST8b: for Dentists	No	No	No	No	No	Yes	1
	CST8c: for Pharmacists	Yes	No	No	Yes	No	Yes	2
	CST8d: for Nurses/Midwives;	No	No	No	No	No	No	0
CST9	Existence of a newsletter/information bulletin/website as a tool for Pharmacovigilance information dissemination	No	No	No	No	No	Yes	1
CST10	Existence of pharmacovigilance advisory committee or an expert committee in the setting capable of providing advice on medicine safety.	Yes	No	No	Yes	Yes	Yes	4

^a^The items in CST6a-e were all considered separately and the answer was found to be No for each item. *UBTH* University of Benin Teaching Hospital Benin-City, Edo State; *UCTH* University of Calabar Teaching Hospital, Calabar Cross-River State; *UPTH* University of Port Harcourt Teaching Hospital, Port Harcourt, Rivers State; *UUTH* University of Uyo Teaching Hospital, Uyo, Akwa- Ibom State; *DELSUTH* Delta State University Teaching Hospital Oghara, Delta State; *NDUTH* Niger Delta University Teaching Hospital Okolobri, Bayelsa State; *ADR* Adverse Drug Reaction; WHO World Health Organization

### Core process indicators

The absolute number of ADR reports received among the 6 hospitals in the previous year ranged from 0 to 26, two hospitals had no reports for the previous year 2015. Furthermore, the total number of reports in the local database ranged from 0 to 831. Cohort event monitoring of antimalarials (artemisinin-based combination therapy) was carried out and completed in UBTH in the five years preceding the analysis as a form of active surveillance. There were limited numbers of reports on ADRs, medication errors, lack of therapeutic effectiveness etc. in most of the centers. Documentation of feedback and causality assessment carried out on reports in the centers was equally poor in this study (Table 3).

**Table 3:** Analysis of WHO Core Pharmacovigilance Process Indicators of the six tertiary hospitals in the South-South zone of Nigeria

Indicator Item	Assessment questions	UCTH	UUTH	UPTH	NDUTH	DELSUTH	UBTH
CP1	Total number of ADR reports received in the previous year	16	0	0	1	9	26
CP2	Reports (current total number) in the local database	41	1	0	12	12	831
CP3	Percentage of total annual reports acknowledged/issued feedback	0	0	0	0	0	0
CP4	Percentage of total reports subjected to causality assessment in the previous year.	0	0	0	0	0	84.6
CP5	Percentage of total annual reports satisfactorily completed and submitted to the local Pharmacovigilance Centre in the previous year.	18.8	0	0	0	77.8	84.6
CP5a	Percentage of reports committed to National Pharmacovigilance Centre database from the local Pharmacovigilance centre	0	0	0	0	0	100
CP6	Percentage of reports of therapeutic ineffectiveness received in the previous year	0	0	0	0	0	0
CP7	Percentage of reports on medication errors reported in the previous year	0	0	0	0	0	7.7
CP8	Percentage of registered pharmaceutical industries having a functional Pharmacovigilance system? Not applicable in this study.	Only applicable at the level of National Pharmacovigilance Centre					
CP9	Number of active surveillance activities initiated, ongoing or completed in the last five years	0	0	0	0	0	1

*UBTH* University of Benin Teaching Hospital Benin-City, Edo State; *UCTH* University of Calabar Teaching Hospital, Calabar Cross-River State; *UPTH* University of Port Harcourt Teaching Hospital, Port Harcourt, Rivers State; *UUTH* University of Uyo Teaching Hospital, Uyo, Akwa- Ibom State; *DELSUTH* Delta State University Teaching Hospital Oghara, Delta State; *NDUTH* Niger Delta University Teaching Hospital Okolobri, Bayelsa State; *ADR* Adverse Drug Reaction; *WHO* World Health Organization

### Core outcome/impact indicators

Unusual reports regarding the development of frequent micturition following use of amlodipine besylate was observed in one of the centers and is being evaluated (Table 4). The number of medicine-related hospital admissions per 1000 admissions ranged from 0.00958/1000 to 1.67/1000 and there were no documentations of medicine related deaths in the death registers in the various hospitals. The documentation of pertinent data was inadequate, rendering calculation of other outcome/impact pharmacovigilance indicators in the institutions difficult (Table 4).

**Table 4:** Analysis of WHO Core Outcome Pharmacovigilance Indicators in six tertiary hospitals in South-South zone of Nigeria^a^

Indicator Item	Assessment questions	UCTH	UUTH	UPTH	NDUTH	DELSUTH	UBTH
CO1	Number of signals generated in the last 5 years	0	0	0	0	0	1^b^
CO2	Number of regulatory notifications issued in the last year	0	0	0	0	0	0
CO3	Number of medicine-related hospital admissions per 1000 admissions^a^	1.67	1.65	0.0985	0.3924	No data	0.97
CO4	Number of medicine-related deaths per 1000 persons served by the hospital per year	Inadequate data	Inadequate data	Inadequate data	Inadequate data	Inadequate data	Inadequate data
CO5	Number of medicine-related deaths per 100,000 persons in the population	Only applicable at the level of National Pharmacovigilance Centre					
CO6	Average cost (US$) of treatment of medicine-related illness	Inadequate data	Inadequate data	Inadequate data	Inadequate data	Inadequate data	Inadequate data
CO7	Average duration (Days) of medicine-related extension of hospital stay	Inadequate data	Inadequate data	Inadequate data	Inadequate data	Inadequate data	5.86 days
CO8	Average cost (US$) of medicine-related hospitalization.	Inadequate data	Inadequate data	Inadequate data	Inadequate data	Inadequate data	Inadequate data

^a^Calculated according to data from Table 1, ^b^ Frequent micturition following use of amlodipine besylate is being evaluated in the centre

*UBTH* University of Benin Teaching Hospital Benin-City, Edo State; *UCTH* University of Calabar Teaching Hospital, Calabar Cross-River State, *UPTH* University of Port Harcourt Teaching Hospital, Port Harcourt, Rivers State; *UUTH* University of Uyo Teaching Hospital, Uyo, Akwa- Ibom State; *DELSUTH* Delta State University Teaching Hospital Oghara, Delta State; *NDUTH* Niger Delta University Teaching Hospital Okolobri, Bayelsa State; *ADR* Adverse Drug Reaction

## Discussion

This is the first published study evaluating the practice of pharmacovigilance in tertiary hospitals of the South-South zone of Nigeria using the WHO indicators. The study has highlighted the strengths and weaknesses of the pharmacovigilance sub-healthcare system in general.

The study revealed that structures were gradually being put in place and there was a general acceptance of the need for pharmacovigilance in all the institutions visited despite institutional challenges. The availability of the newly developed Nigerian national pharmacovigilance policy in some of the centers is a testament to the will of the Nigerian government to institutionalize patient safety through good pharmacovigilance practice.

It was observed that the UBTH performed better than the other hospitals within the zone, this was ascribed to the activities of the pharmacovigilance team and system that started off in the early 90s [2] and has been largely sustained by the commitment of the pharmacovigilance committee, staff and management. It was also observed that despite DELSUTH and NDUTH being relatively smaller hospitals in terms of bed complement, they still performed better than some larger hospitals. This suggests that interest of the key stakeholders in the pharmacovigilance program is needed to sustain the development of the pharmacovigilance system.

Interestingly, one of the centers (DELSUTH) modified the ADR form showing their own hospital logo and domiciling the ADR form to their setting. This showed the willingness of the centre to improve patient safety through a sense of ownership. The inclusion of health facilities in the Nigerian national pharmacovigilance policy was to increase their participation in the pharmacovigilance activities [6]. The study revealed poor budgeting for pharmacovigilance as only a center (UBTH) had financial provision for pharmacovigilance. This was distinct from the finding in Rwanda using the Indicator based pharmacovigilance assessment tool (IPAT) where the hospitals studied had budgetary allocation for pharmacovigilance [31]. The availability of relevant staff and committees are paramount to the development of pharmacovigilance and the hospitals with developed committees and personnel disposition had slightly better reports. It is important to fund pharmacovigilance as development of active pharmacovigilance programs, provision of training, feedback, information dissemination and maintenance of the centers are useful tools in pharmacovigilance that require adequate finances [32]. Capacity development is required for the growth of pharmacovigilance as shown in the review of three countries where insufficient manpower contributed to poor development of pharmacovigilance [27].

The processes and outcomes were however poor in all the facilities probably due to lack of awareness of measuring indices to monitor and evaluate pharmacovigilance. Again, the pharmacovigilance system in this setting is still in their infancy and the requisite culture to ensure effective operations yet to be established. However, it was noted that a cohort event monitoring of antimalarials (artemisinin-based combination therapy) was conducted in UBTH as a part of a national program. This active surveillance of medicines used in a disease of public health importance is useful in obtaining better insights into the safety and tolerability pattern in our setting [4]. The need for the indicators could also be seen in a review of three national centers India, Uganda and South Africa using the WHO minimum requirements for a functional pharmacovigilance system by Maigetter et al. [27] which suggested a more efficient and systematic monitoring for pharmacovigilance system. An awareness of regular pharmacovigilance evaluations with pharmacovigilance indicators would translate to better pharmacovigilance processes and outcomes.

The poor record keeping in all the facilities also made computations of the process and outcomes indicators difficult to achieve. The documentation of medicine related events especially adverse drug reactions were equally poor in this study, this contributed to lack of data even in hospitals where the international coding of diseases was been done. This is not different from what has been reported in other studies about under-recognition of adverse drug reactions and drug related events [33, 34]. It is imperative to inculcate a more articulate approach to routine data gathering and documentation into the healthcare system. Furthermore, planned prospective data collection processes should be put in place to enable evaluation of the outcomes and impact of pharmacovigilance activities.

In the utilization of the WHO pharmacovigilance indicators, it is evident that the scope of reportable incidents by the facilities have been broadened and it is hoped that with the implementation framework of the Nigerian national pharmacovigilance policy, there would be a wider dissemination of the roles that tertiary hospitals are to play in the promotion of pharmacovigilance. The WHO pharmacovigilance indicators would be useful in assessing other tertiary hospitals as it would enable the hospital management develop a strategy towards improving patient safety through pharmacovigilance. It may also help identify areas that need urgent intervention or modification in the health information system management of the tertiary hospitals especially since it is recommended that the indicators be reapplied as needed in the facilities.

### Limitations

The WHO indicators have proven to be quite useful in this assessment. However, absence of trained pharmacovigilance personnel hindered the provision of results for the pharmacovigilance process indicators in the centers. Of note is the limitation of the structural pharmacovigilance indicators to fully capture the functionality of the pharmacovigilance system. Furthermore, the overall poor documentation in all centers limited the derivation of the indicators. Again the derivation of the outcome/impact indicator required in-depth survey which young pharmacovigilance systems are unable to execute. There might be a need to develop a scoring system to quantify the indices thus highlighting the deficiencies in numerical terms.

## Conclusion

This study has shown an urgent need to strengthen the pharmacovigilance systems in the South-South zone of Nigeria. The WHO pharmacovigilance indicators have been proven to be helpful in assessing the pharmacovigilance system in the zone. Improvement in medical record documentation as well as increased institutionalization of pharmacovigilance may be the first steps to improve pharmacovigilance activities in the tertiary hospitals.

## Additional file

Additional file 1: Assessment of the state of Pharmacovigilance in the South-South Zone of Nigeria using WHO Pharmacovigilance indicators. WHO Core Pharmacovigilance Indicators including changes made to phrasing of the assessment questions. (PDF 347 kb)

## Abbreviations

ADR: Adverse drug reaction
DELSUTH: Delta State University Teaching Hospital Oghara, Delta State
ICSR: Individual case safety report
IPAT: Indicator based Pharmacovigilance Assessment Tool
NAFDAC: National Agency for Food, Drugs Administration and Control, Nigeria
NDUTH: Niger Delta University Teaching Hospital Okolobri, Bayelsa State
NPC: National Pharmacovigilance Centre
UBTH: University of Benin Teaching Hospital Benin-City, Edo State
UCTH: University of Calabar Teaching Hospital, Calabar Cross-River State
UPTH: University of Port Harcourt Teaching Hospital, Port Harcourt, Rivers State
UUTH: University of Uyo Teaching Hospital, Uyo, Akwa- Ibom State
WHO: World Health Organization
