# Supplementary material for: Assessment of the state of pharmacovigilance in the South-South zone of Nigeria using WHO pharmacovigilance indicators
Source: BMC Pharmacol Toxicol. 2018 May 31;19:27. doi: 10.1186/s40360-018-0217-2 (PMC5984375; doi:10.1186/s40360-018-0217-2)
Supplement: Supplementary file 1 — Assessment of the state of Pharmacovigilance in the South-South Zone of Nigeria using WHO Pharmacovigilance indicators. WHO Core Pharmacovigilance Indicators including changes made to phrasing of the assessment questions. (PDF 347 kb) [file 40360_2018_217_MOESM1_ESM.pdf]

DRUG SAFETY

Assessment of the state of Pharmacovigilance in the South-South Zone of Nigeria using WHO Pharmacovigilance indicators.

Abimbola O. Opadeyi,[1,2] Annie Fourrier-Réglat [3,4,5] Ambrose O. Isah,[1,2,6]

1. **Department of Clinical Pharmacology and Therapeutics, University of Benin, Benin-City, Edo State, Nigeria.**
2. **Department of Medicine, University of Benin Teaching Hospital, Benin-City, Nigeria.**
3. Univ. Bordeaux, Inserm, Bordeaux Population Health Research Center, team, **Pharmacoepidemiology, UMR 1219, F-33000 Bordeaux, France**
4. **Bordeaux PharmacoEpi, INSERM CIC1401, F-33000 Bordeaux, France**

5 CHU de Bordeaux, Pôle de santé publique, Service de Pharmacologie médicale, F-33000 Bordeaux, France

6. Chairman, National Drug Safety Advisory Committee National Agency for Food and Drug Administration and Control, Federal Ministry of Health. Abuja, Nigeria.

Correspondence
Dr. Abimbola O. Opadeyi,
Department of Clinical Pharmacology and Therapeutics,
University of Benin,
Benin-City,
Edo State,
Nigeria.
felabimbola@yahoo.com
+2348037075435
ORCID NO: 0000-0003-0630-2117

Electronic Supplementary material I

WHO Core Pharmacovigilance Indicators including changes made to phrasing of the assessment questions.

#	Core Structural Indicators - Assessment questions	Changes made to assessment questions for Core Structural Indicators (as applicable)
**CST1**	Is there a Pharmacovigilance Centre / Department / Unit with a standard accommodation?	No changes
**CST2**	Is there a statutory provision (national policy, legislation) for Pharmacovigilance?	Do you have the national pharmacovigilance policy document?
**CST3**	Is there a Drug Regulatory Authority/Agency?	Is there Drug Therapeutic Committee in the hospital?.
**(CST4**	Is there any regular financial provision (e.g. statutory budget) for the Pharmacovigilance centre?	No changes
**CST5**	Has the Pharmacovigilance Centre human resources to carry	No changes.

	out its functions properly?	
**CST6**	Is there a standard ADR reporting form in the hospital?	No changes
	CST6a: Are there relevant fields in the standard ADR form to report suspected medication errors?	No changes.
	CST6b: Are there relevant fields in the standard ADR form to report suspected counterfeit / substandard medicines?	No changes.
	CST6c: Are there relevant fields in the standard ADR form to report therapeutic ineffectiveness?	No changes.
	CST6d: Are there relevant fields in the standard ADR form to report suspected misuse, abuse and/or dependence on medicines?	No changes.
	CST6e: Is there a standard ADR reporting form for general public?	No changes
**CST7**	Is there a process in place for collection, recording and analysis of ADR reports?	No changes.
**CST8**	Is Pharmacovigilance incorporated into the national curriculum of the various health care professions?	Is pharmacovigilance incorporated into the orientation programme curriculum of newly employed health professionals

	CST8a: Is Pharmacovigilance incorporated into the national curriculum of Medical doctors?	- Is pharmacovigilance incorporated into the orientation programme curriculum of newly employed Doctors?
	CST8b: Is Pharmacovigilance incorporated into the national curriculum of Dentists?	Is pharmacovigilance incorporated into the orientation programme curriculum of newly employed Dentists?
	CST8c: Is Pharmacovigilance incorporated into the national curriculum of Pharmacists?	Is pharmacovigilance incorporated into the orientation programme curriculum of newly employed Pharmacists?
	CST8d: Is Pharmacovigilance incorporated into the national curriculum of Nurses/Midwives?	Is pharmacovigilance incorporated into the orientation programme curriculum of newly employed Nurses/Midwives?
	CST8e: Is Pharmacovigilance incorporated into the national curriculum of others-*to be specified*?	Is pharmacovigilance incorporated into the orientation programme curriculum of newly employed others?
**CST9**	Is there a newsletter/information bulletin/website (a tool for Pharmacovigilance information dissemination?)	No changes
**CST10**	Is there a national ADR or pharmacovigilance advisory committee or an expert committee in the setting capable of providing advice on medicine safety?	No changes
	**Core Process Indicators - Assessment questions**	**Changes made to assessment questions for core process indicators (as applicable)**
**CP1**	What is the total number of ADR reports received in the	No changes

	previous year?	
	CP1a: What is the total number of ADR reports received in the previous year per 100,000 persons in population?	
**CP2**	How many reports are (current total number) in the national/regional/local database?	How many reports are in the local database?
**CP3**	What is the percentage of total annual reports acknowledged/ issued feedback?	No changes
**CP4**	What is the percentage of total reports subjected to causality assessment in the year?	No changes
**CP5**	What is the percentage of total annual reports satisfactorily completed and submitted to the National Pharmacovigilance Centre in the previous year?	What is the percentage of total annual reports satisfactorily completed and submitted to the Local Pharmacovigilance Centre in the previous year?
	CP5a: Out of the reports satisfactorily completed and submitted to the National PV Centre, what is the percentage of reports committed to WHO database?	Out of the reports satisfactorily completed and submitted to the Local PV Centre, what is the percentage of reports committed to National PV Centre?
**CP6**	What is the percentage of reports of therapeutic ineffectiveness	No changes

	received in the previous year?	
**CP7**	What is the percentage of reports on medication errors reported in the previous year?	No changes
**CP8**	What is the percentage of registered pharmaceutical industries having a functional Pharmacovigilance system?	Not applicable at this level.
**CP9**	How many active surveillance activities are or were initiated, ongoing or completed the last five years?	No changes
#	**Outcome/Impact Indicators - Assessment questions**	**Changes made to Outcome/Impact indicators assessment questions**
**CO1**	How many signals were generated in the last 5 years by the Pharmacovigilance Centre?	No changes
**CO2**	How many regulatory actions were taken in the preceding year consequent on National Pharmacovigilance activities?	How many regulatory notifications were received from the National PV Centre and how many were disseminated to the health care professionals.

	CO2a: how many Product Label changes (variation)?	Follow on from CO2
	CO2b: how many safety warnings on medicines to: (CO2bi) health professionals (CO2bii) general public?	Follow on as for CO2: (i) and (ii)
	CO2c: how many withdrawals of medicines?	No changes
	CO2d: how many other restrictions in use of medicines?	No changes
**CO3**	What is the number of medicine-related hospital admissions per 1,000 admissions?	No Changes
**CO4**	What is the number of medicine-related deaths per 1,000 persons served by the hospital per year?	No changes
**CO5**	What is the number of medicine-related deaths per 100,000 persons in the population?	Not applicable at the institutional level

**CO6**	What is the average cost (US$) of treatment of medicine-related illness?	Omitted in this study as it would require a cost of illness study as suggested by the indicator manual.
**CO7**	What is the average duration (Days) of medicine-related extension of hospital stay?	No changes.
**CO8**	What is the average cost (US$) of medicine-related hospitalization?	No changes
